# Integrating the Extended Theory of Planned Behavior With the Stages of Change to Predict Exercise Among Chinese People With Type 2 Diabetes

**DOI:** 10.3389/fpubh.2021.772564

**Published:** 2021-11-19

**Authors:** Min Gao, Ping Chen, Xinying Sun, XingLin Feng, Edwin B. Fisher

**Affiliations:** ^1^School of Public Health, Peking University Health Science Center, Beijing, China; ^2^Nuffield Department of Primary Care Health Sciences, University of Oxford, Oxford, United Kingdom; ^3^Department of Health Policy and Management, School of Public Health, Peking University, Beijing, China; ^4^Department of Health Behavior, Gillings School of Global Public Health University, Chapel Hill, NC, United States

**Keywords:** exercise, physical activity, type 2 diabetes, extended theory of planned behavior, stages of change, self-identity, descriptive norm constructs

## Abstract

**Background:** There have been very limited prospective studies examining social-cognitive models within stages of behavior change in the exercise domain.

**Purpose:** We examined the utility of the theory of planned behavior (TPB), incorporating self-identity and descriptive norm constructs, to predict exercise behavior across the stages of change, in individuals with type 2 diabetes.

**Methods:** Data were obtained from a longitudinal study. Multi-group structural equation modeling was used to estimate the association between extended TPB constructs and exercise within different stages groups.

**Results:** 647 individuals completed a self-report questionnaire at baseline and at 3 months follow-up. The extended TPB model explained 8–15% variance of exercise behavior and 42–81% variance of exercise intention within three stages groups in the cross-sectional design. The extended TPB model explained 4%-13% variance of exercise behavior and 42–66% variance of exercise intention in the longitudinal design. Intention was significantly related to exercise behavior in the pre-action and action stages. Self-identity, perceived behavioral control and descriptive norms were stronger predictors of intention in different stages.

**Conclusion:** Discontinuity patterns in the extended theory of planned behavior for the different stages groups were found. Intention was a significant predictor of exercise in the pre-action and action stages at 3 months.

## Introduction

Over the past three decades, the prevalence of diabetes has increased in China from <1% in 1980 to 12.8% in 2017 (1). Exercise is recommended for all individuals with diabetes as a part of the management of glycemic control (2–4). However, many studies have shown that the percentage of Chinese patients with type 2 diabetes who start exercising is lower (5, 6). It is crucial that the determinant factors of exercise activity among people with type 2 diabetes be explored and that effective interventions be promoted.

Ajzen's theory of planned behavior (TPB) has been widely applied to and discussed for interventions involving exercise (7–11). The TPB includes four main constructs: attitude, subjective norm, perceived behavioral control (PBC), and intention. It is hypothesized that the intention is a function of attitude toward the behavior, subjective norm and PBC (12). The theory has been used extensively in the prediction of exercise in people with type 2 diabetes (13–15). One study in Australia found that in patients with type 2 diabetes, intention could explain 28% of the variance in exercise, and attitude, subjective norm, and PBC could explain 73% of the variance in intention (16). TPB was popular due to its ability to explain and predict and its operability of how to measure the variables (17).

Many previous studies have applied TPB to explain and predict a wide range of behaviors (7, 18–20). And it has been found that there were some limitations of TPB. Firstly, it is insufficient for TPB to explain a complicated psychological mechanism. So many studies modified TPB to add important factors to explain or predict specific behaviors (21–23). And Ajzen further suggested that the TPB was open to further expansion if additional predictors could be identified that explained more variance when included (24). Secondly, it was found that the past behavior contributed an extra 19% variance to the prediction of exercise controlling for other TPB variables (25). And it has been indicated that TPB had less power in longitudinal design comparing with cross-sectional design (7). Thirdly, TPB has been demonstrated different efficacy on different behavior types which means only one behavior would be focused on. A systematic review of 237 prospective studies showed that physical activity was better predicted among different behavior types (7). Finally, TPB focused on rational things but excluded the influence of unconsciousness and the role of emotion (12).

In order to make up for these limitations of TPB, we added two another factors self-identity and subjective norms to help explain and predict exercise. Self-identity can be conceptualized as the salient aspects of an individual's sense of self that are related to certain behaviors (26). For example, people who strongly view themselves as physically fit individuals are more likely to participate in physical activities. Increasingly, studies are finding that inclusion of self-identity explains more variance in behavioral intention, after accounting for traditional TPB constructs (27–29). A meta-analysis of 40 independent studies explored the role of self-identity in the TPB and found that the inclusion of self-identity could explain 6% additional variance in intentions (30).

Subjective norms can be described as the individual's perceptions of what important others think ought to be done (injunctive norms) (31). Ajzen and Fishbein suggested that the subjective norm construct should also include the individual's perceptions of what important others do (descriptive norms), in addition to injunctive norms. One meta-analysis found that the relationship between descriptive norm and behavioral intention was stronger in health-promoting behaviors, such as exercise (32). However, the implications for exercise interventions are limited, because the meta-analysis only included two studies on exercise behavior, and both studies included only college students.

It has been indicated that TPB was unsuitable for longitudinal study due in part to the fact that it would be influenced severely by past behavior. So in this study, the stages of change model was integrated with the extended TPB (30). The stages of change model could assess the current status of behavior. It is expected that TPB could show better predictive ability when people are separated according to different stages other than regarded as a whole.

The stages of change (30) model proposes that there are five stages of change: pre-contemplation, contemplation, preparation, action, and maintenance. People at different stages will think and behave in qualitatively different ways (33). A limited number of studies have explored the application of the TPB to the prediction of stage-specific patterns of physical activity. These studies have found that the magnitude of the effect size of social-cognitive variables on behavior was different in different stages of behavior (34, 35).

Therefore, this study aimed to (a) test the validity of an extended version of the TPB, which included self-identity and descriptive norm, for predicting exercise, and (b) examine the stage-specific effects of the extended TPB on exercise behavior using the stages of change model.

## Materials and Methods

### Study Design and Setting

This is a longitudinal study. The participants were recruited by cluster sampling from 22 community health centers in Beijing (in the Shunyi and Tongzhou districts) from July to November in 2018 (36).

Questionnaire survey was conducted at baseline and at 3-month follow-up through one-to-one interviews by well-trained investigators. Every investigator was trained before every investigation through standard lessons by the same trainer and investigators were asked to simulate the inquiry with each other.

At each health center, a team of physicians and nurses was responsible for the recruitment of participants, and collecting blood samples and biomarkers (including height, weight, waist circumference, and blood pressure). Well-trained investigators conducting face-to-face interviews for questionnaire investigation.

### Participants

Inclusion criteria were as follows: aged between 18 and 70 years; hemoglobin A1c (HbA_1c_) ≥7.5% (as a measure of glucose control); permanent residence in Shunyi or Tongzhou district; without severe intellectual disability, Alzheimer's disease, or other mental disorders; not participating in other scientific studies; and agreeing to participate. Exclusion criteria included severe diabetes complications or other severe diseases.

There were 819 potential participants at baseline. According to the requirements for calculating sample size of structural equipment model (37), the degree of freedom of the structural equation model can be calculated as 204. When α = 0.05, β = 0.1 and RMSEA reached 0.05, the required sample size was 133. So the participants were enough. Of those 819 potential participants, 665 people were followed after 3 months. Individuals who did not provide sufficient information on demographic and lifestyle factors were excluded from the study (*n* = 18). Data from the 647 individuals were included in the final analysis. This study was conducted in accordance with the Declaration of Helsinki. All participants provided prior written informed consent. Approval for the study was obtained from Medical Ethics Committee of Peking University (IRB00001052-17044).

### Measurement

#### Covariates

This data was obtained from a longitudinal study which divided the participants into two groups of intervention and control group. The difference between the groups lies in the doctor-patient relationship. The doctor-patient relationship at each community health station was categorized into two categories: directive support (the doctor assumes most of the responsibility for the management of the patient's condition and tells patients how they should do) and nondirective support (the doctor cooperates with patients and accepts their advice) (38). Moreover, stages of diet behavior (precontemplation, contemplation, preparation, action, and maintenance) and stages of medication behavior (precontemplation, contemplation, preparation, action, and maintenance) were also assessed and were considered into covariates.

Other variables were gender (women and men), age (≤50, 51–55, 56–60, 61–65, and ≥66), marital status (married and single), education level (primary school and below, middle school, high school, and university and above), and household monthly income per person (<3,000, 3,000–3,999, 4,000–4,999, 5,000–9,999, ≥10,000). Smoking status was classified into three categories: current smoker, past smoker, and nonsmoker. Drinking status was also classified into three categories: current drinker, past drinker, and nondrinker. Body mass index (BMI) was calculated by height and weight measurements. Diabetes duration was calculated as current year minus age at diagnosis. Hemoglobin A1c (%) at baseline was treated as a continuous variable.

#### Self-Reported Exercise

Self-reported exercise was measured with the Chinese version of the International Physical Activity Questionnaire at baseline and 3 months. The questionnaire was used to collect data on the frequency of engagement in common high-, moderate-, and low- intensity exercises (39). Durations of these different intensities of exercise were set for at least 10 min per bout. The equation for the amount of exercise was as follows:

*Total metabolic equivalents (METs)* = *METs (activities of moderate intensity were defined as 4 METs; activities of vigorous intensity were defined as 8 METs)* × *days of exercise per week* × *duration for exercise per day*.

#### Stages of Change

Stage of change was assessed with five-category measure in health behavior participation, which was a one-item questionnaire developed by Prochaska and Marcus (40). Participants were classified into one of five stages: (1) pre-contemplation (do not participate in exercise and do not intend to start), (2) contemplation (do not participate in exercise but are considering starting), (3) preparation (do not participate in exercise but plan to start soon); (4) action (are currently participating in exercise), and (5) maintenance (have participated in exercise for 6 months or more). The questionnaire was used to collect data on stages of exercise, diet, and medication behaviors.

#### Extended TPB Questionnaire

A questionnaire based on the variables of extended TPB was designed for this study. The questionnaire was designed to reflect Chinese culture and the current social situation. We designed several items for each dimension. Participants were allowed to choose the options of 5 degrees from “totally disagree” to “totally agree.”

The dimension of intention reflected the intention to exercise such as “I have planned when to exercise regularly.” The items of attitude focus on whether the attitude to exercise is positive, such as “Exercise is very effective for controlling blood sugar.” Self-identity means whether the participants think they are fit to exercise, such as “I think I am good at exercising.” Subjective norm reflects the opinion of someone else important, such as “People who are important to me think that I should exercise regularly.” And descriptive norm was conceptualized as the perception of what important others do, such as “Many friends with T2D are exercising regularly.” PBC were conceptualized as questions like “I've figured out how to solve the problems I might encounter during exercise.” The specific items are presented in Appendix, Table A.

Reliability and validity for the measurement of self-identity, subjective norm, descriptive norm, intention, and perceived behavioral control are presented in Appendix, Table A. Cronbach's alpha values for each factor were as follows: intention (0.960), attitude (0.747), self-identity (0.750), subjective norm (0.63), descriptive norm (0.621), and PBC (0.653).

### Data Analysis

Due to the limited number of participants in the pre-contemplation, contemplation, and preparation stages at baseline, we grouped these individuals into a pre-action group. Descriptive statistics (frequencies, proportions, means, and standard deviations) within three stages were examined. The chi-square test and one-way analysis of variance were used to estimate the differences across three stages. Variables were tested for skewness and kurtosis, and the differences were measured with the Kruskal-Wallis test if not normally distributed. Factor analysis was used to measure construct validity, and Cronbach's alpha was used to measure internal consistency. The correlations between subscales in the cross-sectional and longitudinal design were estimated. Structural equation modeling was conducted in the total population to predict exercise intention and exercise. The baseline model (baseline extended TPB constructs and baseline exercise) and the full model (baseline extended TPB constructs and exercise behavior at follow-up) were tested among participants in baseline stage groups. A maximum likelihood estimator was used to construct and fit the two models. Data were analyzed with STATA 14.0 (StataCorp LLC, College Station, TX, USA), and structural equation models were constructed using Mplus 7.0 (Muthén & Muthén, Los Angeles, CA, USA).

## Results

### Stage-Based Demographic Characteristics and Exercise

Stage-based differences in demographic variables are shown in Table 1. Compared at baseline and at 3 months, differences in exercise volume were significant across stages (*P* < 0.001). At baseline, participants in the maintenance (mean = 17.80 [SD = 43.42]) and action stages (mean = 12.36 [SD = 28.71]) had significantly higher exercise volume than participants in the pre-action stage (mean = 0.46 [SD = 1.71]).

**Table 1:** Change of exercise amount and baseline demographic characteristics across the stages of change.

	**Total**	**Pre-action**	**Action**	**Maintain**	**X^**2**^/F**
**Exercise, Mean(SD)** (MET-hours/week)					
Baseline	13.07 (34.63)	0.46 (1.71)	12.36 (28.71)	17.80 (41.01)	12.70^***^
3 months	14.01 (37.52)	1.23 (3.05)	12.05 (34.91)	19.20 (43.42)	11.89^***^
**Gender (%)**					2.89
Female	51.62	51.88	57.89	49.34	
male	48.38	48.12	42.11	50.66	
**Age group (%)**					13.39
≤50	11.13	15.79	12.03	9.19	
51–55	15.15	17.29	11.28	15.75	
56–60	21.02	24.81	20.30	19.95	
61–65	28.90	27.07	32.33	28.35	
≥66	23.80	15.04	24.06	26.77	
**Education level (%)**					2.59
Primary school and below	10.66	12.03	13.53	9.19	
Middle school	45.90	45.11	45.11	46.46	
High school	26.28	25.56	26.32	26.51	
University and above	17.16	17.29	15.04	17.85	
**Marital status (%)**					0.80
Single	6.65	8.27	6.77	6.04	
Married	93.35	91.73	93.23	93.96	
**Household monthly income per person(%)**					4.31
<3,000	29.83	29.32	33.83	28.61	
3,000–3,999	29.83	30.08	28.57	30.18	
4,000–4,999	14.22	13.53	17.29	13.39	
≥5,000	26.12	27.07	20.30	27.82	
**Stage of diet behavior (%)**					65.00^***^
Precontemplation	35.24	38.35	27.07	37.01	
Contemplation	9.58	15.04	9.02	7.87	
Preparation	3.86	9.77	3.76	1.84	
Action	7.57	6.02	19.55	3.94	
Maintenance	43.74	30.83	40.60	49.34	
**Stage of medication behavior (%)**					14.96
Pre-contemplation	8.50	6.02	6.02	10.24	
Contemplation	0.77	0.00	1.50	0.79	
Preparation	0.31	0.00	0.75	0.26	
Action	3.55	3.76	7.52	2.10	
Maintenance	86.86	90.23	84.21	86.61	
**Smoke situation (%)**					11.80^*^
Current smoker	23.96	32.33	17.29	23.36	
Past smoker	15.46	9.02	16.54	17.32	
Nonsmoker	60.59	58.65	66.17	59.32	
**Drink situation (%)**					3.72
Current drinker	29.68	23.31	30.08	31.76	
Past drinker	7.88	9.77	7.52	7.35	
Nondrinker	62.44	66.92	62.41	60.89	
**BMI, Mean (SD)**	26.70 (4.07)	27.78 (4.71)	26.35 (3.11)	26.44 (4.08)	6.03^**^
**Diabetes duration, Mean(SD)**	4.83 (3.53)	5.10 (3.80)	4.57 (2.66)	4.83 (3.69)	0.74
**HbA_1c_ at baseline, Mean (SD)**	7.12 (1.25)	7.45 (1.49)	7.05 (1.14)	7.04 (1.19)	5.75^**^
**Doctors-patients relationship (%)**					1.78
Directive support	34.93	39.10	33.83	33.86	
Nondirective support	33.69	33.08	32.33	34.38	
N	647	133	133	381	

*Notes:* ①

* *P < 0.05;*

** *P < 0.01;*

*** *P < 0.001*.

② *Total Metabolic Equivalents (METs), METs (moderate intensity activities were defined as 4 METs, vigorous intensity activities were defined as 8 METs) × days of exercise per week × duration for exercise per day*.

At baseline, significant stage-based differences were observed for dietary behavior (*P* < 0.001), smoking status (*P* < 0.05), BMI (*P* < 0.01), and HbA_1c_ at baseline (*P* < 0.01). Across all exercise behavior stages, 48.68% of participants had not started diet control, whereas 90.41% had already taken medication. Most people did not smoke (60.59%) or dink (62.44%). Participants in the pre-action stage had higher BMIs (mean = 27.78 [SD = 4.71]), longer diabetes durations (mean = 5.10 [SD = 3.80]), and higher HbA_1c_ levels (mean = 7.45 [SD = 1.49]).

### Scores of Extended TPB Constructs

The means and standard deviations for each construct across the three stages of change are presented in Table 2. The scores of all constructs in the advanced stages of exercise were significantly higher than those in the less advanced stages. Scores of all constructs in the pre-action stage were the lowest compared with those in the other stages.

**Table 2:** Means (standard deviations) for the extended TPB across the stages of change at baseline.

	**Total**	**Pre-action**	**Action**	**Maintain**	**Chi^**2**^**	***P***
Intention	39.67 (12.79)	25.26 (13.72)	43.60 (9.38)	43.33 (9.53)	151.04	<0.001
Attitude	22.04 (3.08)	21.30 (3.52)	22.24 (3.03)	22.23 (2.90)	7.22	0.027
Self-identity	18.11 (4.31)	15.37 (4.83)	18.51 (4.43)	18.92 (3.64)	61.22	<0.001
Subjective norm	11.78 (3.33)	10.58 (4.11)	12.02 (3.19)	12.11 (2.98)	13.97	<0.001
Descriptive norm	10.87 (3.12)	9.89 (3.58)	11.23 (2.83)	11.08 (2.98)	12.42	<0.001
PBC	15.84 (4.75)	12.74 (5.14)	16.63 (4.32)	16.65 (4.30)	59.63	<0.001

*All differences based on Kruskal-Wallis test. PBC, perceived behavior control*.

### Cross-Sectional Structural Equation Modeling to Explain Exercise

Multi-group structural equation modeling was used to estimate the internal correlations of exercise and components of the extended TPB in the three stages at baseline and at 3 months follow-up. Pearson correlations between the TPB measures between baseline and 3 months were estimated (Appendix, Table B). Goodness-of-fit indexes of multilevel structural equation models at baseline and at 3 months are presented, along with other detailed information, in Appendix, Table C. As is shown in Table 3, the baseline model accounted for 8, 15, and 11% of the variance of behavior in the pre-action, action, and maintenance stages. Explained variance in intention were 81, 42, and 49% in the pre-action, action, and maintenance stages. Intention was significantly related to exercise in the action (β = 0.20, *P* < 0.05) and maintenance (β = 0.15, *P* < 0.001) stages. Self-identity (β = 0.56, *P* < 0.001 in the action stage, β = 0.34, *P* < 0.001 in the maintenance stage), PBC (β = 0.54, *P* < 0.01 in the pre-action stage, β = 0.40, *P* < 0.001 in the maintenance stage), and descriptive norm (β = 0.15, *P* < 0.05 in the maintenance stage) were stronger predictors of intention.

**Table 3:** Multiple regression analysis predicting behavioral intention and behavior across the stages.

	**Baseline model**			**The full model**		
	**Pre-action**	**Action**	**Maintain**	**Pre-action**	**Action**	**Maintain**
**Exercise**						
R^2^	0.08	0.15	0.11	0.13	0.12	0.04
Intention	−0.02	0.20^**^	0.15^*^	0.33^*^	0.13^*^	0.12
PBC	0.23	−0.03	0.09	−0.12	0.05	−0.04
**Intention**						
R^2^	0.81	0.42	0.49	0.66	0.42	0.53
Attitude	0.18	−0.11	−0.02	0.08	−0.16	−0.03)
Self-identity	0.03	0.56^***^	0.34^***^	0.18	0.56^***^	0.37^***^
Subjective norm	0.46	0.09	0.07	0.15	0.08	0.07
Descriptive norm	0.14	0.13	0.15^*^	0.16	0.12	0.14^*^
PBC	0.54^**^	0.11	0.40^***^	0.61^**^	0.17	0.42^***^

* ①*P < 0.05;*

** *P < 0.01;*

*** *P < 0.001. PBC, perceived behavior control*.

② *The models were adjusted for gender, age groups, marital status, education level, household monthly income per person, stage of diet behavior and medication behavior, smoke situation, drink situation, BMI, diabetes duration, HbA_1c_ (%) at baseline and doctors-patients relationship*.

### Structural Equation Modeling to Predict Exercise at Follow-Up

The full model explained 13, 12, and 4% of the variance of behavior, 66 and 42%, and 53% of the variance of intention across stages at 3 months. Baseline intention was significantly related to exercise in the pre-action (β = 0.33, *P* < 0.05) and action stages (β = 0.13, *P* < 0.05). No association between baseline PBC and exercise across the three stages was observed (*P* > 0.05). Self-identity was associated with intention in the action (β = 0.56, *P* < 0.001) and maintenance stages (β = 0.37, *P* < 0.001). PBC could predict intention in the pre-action (β = 0.61, *P* < 0.01) and maintenance stages (β = 0.42, *P* < 0.001). Descriptive norm could predict intention in the maintenance stage (β = 0.14, *P* < 0.05).

## Discussion and Conclusion

### Discussion

#### Main Findings

This study provided evidence of the applicability of an extended TPB that adds self-identity and descriptive norm in the longitudinal design about exercise for patients with diabetes. Most importantly, a discontinuity pattern was observed across stages of exercise within the extended constructs of the TPB. Intention was a strong predictor of exercise. Self-identity, PBC, and descriptive norm were strong predictors of intention in different stages.

#### Extended TPB Could Predict Exercise in Longitudinal Studies

The explained variance for exercise at 3 months did not decline comparing with that at baseline, which provides evidence of the long-term utility of the extended TPB model integrating stages of change model. TPB showed better efficacy to predict exercise among different behavior types, and a meta-analysis found that TPB could explain 23.9% variance (7). It seemed that the degree of interpretation in this meta-analysis was higher than that in this study, but the type of study should be taken into consideration. Those studies included in meta-analysis were cross-sectional design. Our results were comparable to many longitudinal studies on the associations between TPB and exercise (41, 42). Plotnikoff demonstrated 8% variance explained by TPB on exercise behavior. As is shown in our results, the degree of explanation for different stages is different. And 8% is between maximum and minimum, which reminded us that the past behavior had influence on the actual behavior. A cohort of 1,427 adults showed that baseline TPB constructs could explain 13% of the variance in exercise behavior over a 15-year period (43). The TPB in the cohort study also integrated other variables into TPB, such as moral norm.

#### Intention Played Different Roles in Different Stages of Change

The results of our study suggest that intention is essential in the prediction of exercise behavior, which is consistent with many studies (7, 44). In the cross-sectional analysis, intention was found to be significantly associated with behavior in the action and maintenance stages. However, in the longitudinal analysis, baseline intention was found to be a significant predictor of exercise over 3 months in the pre-action and action stages. Participants who have exercised for at least 6 months at baseline may have already developed an exercise routine, and their baseline intention to exercise could be underestimated. However, for patients who were in the pre-action stage at baseline, after 3 months, their intention has a relatively larger effect on exercise compared with patients in the action stage before. It may be that in people who had not started exercising before, the impact of intention on their behavior when they do start exercising is more significant compared with that of people who were exercising at baseline. This explanation is supported by the findings of one previous longitudinal study (45). PBC is another well-discussed predictor of behavior and intention that reflects one's ability to reduce the impact of other inhibiting factors, such as weather, environment, time, and effort (46). In our study, PBC can't predict exercise in all stages of exercise at 3 months. Many previous studies have found PBC to be more strongly associated with intention than with behavior (47, 48). It is possible that participants who have high levels of confidence are more likely to have good intentions to be physically active and that these intentions do not necessarily translate into action. However, in our study, PBC was strongly associated with intention in the pre-action and maintenance stages. PBC reflected one's ability to reduce the impact of other inhibiting factors.

#### Variables of Extended TPB Contributed to Intention and Actual Behavior

In line with many studies (29, 49, 50), we found self-identity to be associated with intention in the action and maintenance stages at baseline and 3-month follow-up, suggesting that individuals who perceived themselves as physically active are more likely to have higher intentions to engage in exercise. There is evidence suggesting that the inclusion of self-identity measures in the TPB could account for additional variance in exercise intentions (26). Our study also found that the impact of self-identity on intention in patients in the action stage is greater than that in patients in the maintenance stage. As indicated in Table 2, people in the maintenance stage at baseline are more likely to identify themselves as physically fit. However, for patients in the action stage, increases in the scores of self-identity would exert greater influence on intention.

Our study found that descriptive norm was significantly associated with intention in the maintenance stage. Important others, such as friends, may have strong social influence on one's behavior (31), and the influence is more significant in the maintenance stage. For patients who continue exercising for more than 6 months, peer support and influence from important others may contribute to the maintenance of exercise participation (32).

Attitude and subjective norm were found to be insignificantly associated with intention across all stages in the cross-sectional and longitudinal analyses, which may be due to the characteristics of our sample. More than 90% of individuals agreed or strongly agreed that exercise offers health benefits, which may have reduced the magnitude of the association between attitude and intention. For subjective norm, 79% of our sample had entered into the action and maintenance stages. They may have perceived less pressure from friends and peers because they had already been engaged in exercise. Many studies have found that subjective norm is a weak and non-significant predictor of intention (51, 52). Previous studies have suggested that subjective norm may be not associated with later stages of exercise because of a lack of instrumental value (53).

### Limitations

Limitations in this study should be acknowledged. First, due to the limited number of participants, we had to combine participants in the pre-contemplation, contemplation, and preparation stages into one stage: the pre-action stage. Therefore, we were unable to analyze the differences among those three stages. Secondly, exercise activity was assessed with a self-reported questionnaire, which may have increased the risk of inaccurate recall. To increase the accuracy of the self-reported data, our investigators were trained to help people with diabetes understand the types and durations of exercise, and to encourage people with diabetes to recall recent exercise activity. Finally, there is a fact that behavior would be influenced by unconsciousness and emotions (54, 55). And it is obvious that perceived social support and environment would also affect behavior. But in this study, these factors were ignored. This point is also the limitation of Theory of Reasoned Action. In the future, researchers could contribute to this part.

### Conclusion

This study has investigated discontinuity patterns across three stages of exercise within the extended TPB. Intention was found to strongly predict exercise in the pre-action and action stage. PBC and self-identity were significant predictors of intention in pre-action and action, respectively. Perceived behavioral control and descriptive norm was significantly associated with intention in the maintenance stage. These findings have underscored the qualitative differences between the stages and may further guide the research and the design of interventions integrating these approaches.

### Practice Implications

Our study provides evidence toward the utility for practitioners and researchers to operationalize exercise interventions for patients with diabetes, using an extended TPB that included self-identity and descriptive norm variables.

Our findings suggest that such interventions would need to promote the importance of exercise intention, with specific emphasis on the enhancement of positive self-identity and strong PBC beliefs in patients in the pre-action and action stages. For patients in the maintenance stage, encouraging self-control and self-recognition may lead to more physical activity. For example, assessing the stage of change before giving interventions. For patients in the pre-action and action stages, design special lectures for them to improve their self-identity and PBC beliefs. But for patients in the maintenance stage, contents of lectures need to focus on tips for maintaining self-discipline and persistence.

## Data Availability Statement

The raw data supporting the conclusions of this article will be made available by the authors, without undue reservation.

## Ethics Statement

All participants provided prior written informed consent. Approval for the study was obtained from Medical Ethics Committee of Peking University (IRB00001052-17044).

## Author Contributions

MG, XYS, and XLF contributed to the conception and design of the work. MG and XYS contributed to the acquisition, analysis, or interpretation of data. EBF guided the investigation. MG and PC drafted the manuscript and are co-first authors. XYS and XLF critically revised the manuscript. All authors contributed to the article and approved the submitted version.

## Funding

This work was funded by the National Natural Science Foundation of China (Grant Nos. 71673009 and 72174008).

## Conflict of Interest

The authors declare that the research was conducted in the absence of any commercial or financial relationships that could be construed as a potential conflict of interest.

## Publisher's Note

All claims expressed in this article are solely those of the authors and do not necessarily represent those of their affiliated organizations, or those of the publisher, the editors and the reviewers. Any product that may be evaluated in this article, or claim that may be made by its manufacturer, is not guaranteed or endorsed by the publisher.
