# Supplementary material for: Integrating the Extended Theory of Planned Behavior With the Stages of Change to Predict Exercise Among Chinese People With Type 2 Diabetes
Source: Front Public Health. 2021 Nov 19;9:772564. doi: 10.3389/fpubh.2021.772564 (PMC8640452; doi:10.3389/fpubh.2021.772564)
Supplement: Supplementary file 1 [file Table_1.DOCX]

**Table A Reliability and construct validity of the questionnaire.**

| Variables | Items | CFA | Cronbach α | % of variance |
| --- | --- | --- | --- | --- |
| Intention | 1. I want to exercise regularly | 0.77 | 0.960 | 30.71 |
|  | 2. I am determined to start exercise regularly | 0.86 |  |  |
|  | 3. I have planned to exercise regularly and prepare to start | 0.86 |  |  |
|  | 4. I have planned when to exercise regularly | 0.87 |  |  |
|  | 5. I have planned where to exercise regularly | 0.83 |  |  |
|  | 6. I have decided what kind of exercise to take | 0.86 |  |  |
|  | 7. I have planned the frequency of regular exercise every week | 0.80 |  |  |
| Attitude | 1. Exercise is very effective for controlling blood sugar | 0.73 | 0.747 | 9.66 |
|  | 2. Exercise can effectively prevent the complications | 0.70 |  |  |
|  | 3. Exercise is very effective for weight control | 0.63 |  |  |
|  | 4. I feel physically comfortable after exercise | 0.59 |  |  |
|  | 5. I feel emotionally comfortable after exercise | 0.57 |  |  |
| Self-identity | 1. I think I am good at exercising | 0.80 | 0.750 | 7.00 |
|  | 2. I think I am in good health | 0.77 |  |  |
|  | 3. I think I exercise regularly | 0.73 |  |  |
|  | 4. People may think I don’t like exercise | 0.64 |  |  |
| Subjective norm | 1. People who are important to me think that I should exercise regularly | 0.89 | 0.763 | 6.92 |
|  | 2. People who are important to me want me to exercise regularly | 0.91 |  |  |
| Descriptive norm | 1. Many friends with T2D are exercising regularly | 0.82 | 0.621 | 4.64 |
|  | 2. Many people are exercising regularly. | 0.79 |  |  |
| PBC | 1. I know what things will interfere with regular exercise | 0.81 | 0.653 | 5.31 |
|  | 2. I've figured out how to solve the problems I might encounter during exercise | 0.78 |  |  |

| **Table B Correlation matrix between baseline extended TPB variables and exercise** | | | | | | | | |
| --- | --- | --- | --- | --- | --- | --- | --- | --- |
|  | 1 | 2 | 3 | 4 | 5 | 6 | 7 | 8 |
| 1.Leisure time exercise at baseline | 1.000 |  |  |  |  |  |  |  |
| 2.Leisure time exercise at 3 months | 0.410^***^ | 1.000 |  |  |  |  |  |  |
| 3.Intention | 0.195^***^ | 0.166^***^ | 1.000 |  |  |  |  |  |
| 4.Attitude | 0.059 | 0.067 | 0.234^***^ | 1.000 |  |  |  |  |
| 5.Self-identity | 0.230^***^ | 0.180^***^ | 0.400^***^ | 0.211^***^ | 1.000 |  |  |  |
| 6.Subjective norm | -0.002 | 0.046 | 0.285^***^ | 0.218^***^ | 0.183^***^ | 1.000 |  |  |
| 7.Descriptive norm | 0.051 | 0.105^**^ | 0.246^***^ | 0.218^***^ | 0.177^***^ | 0.236^***^ | 1.000 |  |
| 8.Perceived behavioral control | 0.071 | 0.068 | 0.592^***^ | 0.222^***^ | 0.245^***^ | 0.250^***^ | 0.168^***^ | 1.000 |
| Notes: ①*: P<0.05; **P<0.01;***: P<0.001. | | | | | | | | |

| **Table C Goodness-of-fit indexes for the three stages subsamples in the**  **cross-sectional and longitudinal design** | | | | | | | | |
| --- | --- | --- | --- | --- | --- | --- | --- | --- |
|  | N | X^2^ | df | X^2^/df | RMSEA | CFI | TLI | SRMR |
| **Baseline model** |  |  |  |  |  |  |  |  |
| Pre-action | 133 | 833.49 | 535 | 1.56 | 0.064 | 0.849 | 0.831 | 0.073 |
| Action | 133 | 858.84 | 531 | 1.62 | 0.064 | 0.831 | 0.808 | 0.071 |
| Maintain | 381 | 753.38 | 530 | 1.42 | 0.033 | 0.944 | 0.936 | 0.054 |
| **The full model** |  |  |  |  |  |  |  |  |
| Pre-action | 133 | 802.40 | 558 | 1.56 | 0.050 | 0.906 | 0.895 | 0.063 |
| Action | 133 | 864.63 | 579 | 1.62 | 0.055 | 0.851 | 0.832 | 0.067 |
| Maintain | 381 | 952.40 | 580 | 1.42 | 0.038 | 0.918 | 0.908 | 0.054 |

Note: RMSEA=root mean squared error of approximation; TLI =Tucker-Lewis Index; CFI=comparative fit index; SRMR=standardized root mean squared residual
